# The impact of vitamin D supplementation on body fat mass in elite male collegiate athletes

**DOI:** 10.1186/s12986-021-00578-9

**Published:** 2021-05-21

**Authors:** Itaru Kawashima, Takashi Tsukahara, Ryosuke Kawai, Takafumi Mizuno, Shinya Ishizuka, Hideki Hiraiwa, Shiro Imagama

**Affiliations:** 1 Department of Orthopaedic Surgery, Nagoya University Graduate School of Medicine, 65 Tsurumai, Showa-ku, Nagoya, Aichi 466-8550 Japan; 2 Department of Orthopaedic Surgery, Asahi University Hospital, 3-23 Hashimotocho, Gifu, Gifu 500-8523 Japan

**Keywords:** Vitamin D, Supplementation, Body fat percentage, Body composition, Male collegiate athletes, COVID-19

## Abstract

**Background:**

Whether vitamin D supplementation has any effect on body fat percentage, especially among elite athletes, remains unclear. The aim of this study was to evaluate the effect of vitamin D supplementation on serum vitamin D level in elite male collegiate athletes and to analyze its effect on body fat percentage.

**Methods:**

We enrolled a total of 42 elite male collegiate athletes in this prospective cohort study. In March 2020, body composition monitoring and blood test were performed. All athletes were provided with vitamin D3 supplement tablets of 25g/day. The use of the supplement was dependent on athletes preference. During the study period, their club activities were stopped for 2months due to the coronavirus disease 2019 outbreak. A second examination, similar to the first one, was performed after approximately 3months. Supplement usage by each athlete was also confirmed. The participants were divided into a non-supplement group (without supplementation, n=15) and a supplement group (with supplementation, n=27).

**Results:**

Regarding baseline data at initial examination, the non-supplement and supplement groups showed significant differences in the mean body fat percentage (9.0% and 12.1%, respectively; *P*=0.03) and serum 25(OH)D level (22.7 and 18.5ng/mL, respectively, *P*=0.02). At the time of the second examination, there were no significant differences in the results of both the groups. In terms of mean change value from the first to the second examination, there were significant differences in body fat percentage (1.9 and 0.2%, respectively, *P*=0.02) and serum 25(OH)D level (1.7 and 7.2ng/mL, respectively, *P*<0.001) between the two groups.

A significant negative correlation was observed between the change ratio of body fat percentage and change value of serum 25(OH)D level (*r*=0.37, *P*=0.02).

**Conclusions:**

Vitamin D supplementation of 25g/day significantly increased the serum 25(OH)D level in elite male collegiate athletes. Vitamin D supplementation may play a role in maintaining athletes body fat percentage under circumstances where sports activity has decreased.

## Background

Vitamin D has proposed roles not only in the inflammatory response, immunity, neuromuscular function, and reduction of incidence of carcinomas but also in bone health. Vitamin D deficiency has been correlated to the risk of bone stress fracture, a common overuse injury in elite athletes [1,5]. Therefore, the importance of adequate vitamin D levels in athletes has become a trending topic of interest [6]. Athletes tend to have an increased risk of vitamin D deficiency; however, it has been reported in the general population as well [7,9]. Vitamin D supplementation seems to have a possibility to improve vitamin D deficiency in athletes. However, the validity of this idea is unknown.

Furthermore, ideal weight control is perceived as an advantage in athletes [10]. Vitamin D level is inversely related to body weight, body mass index (BMI), and body fat percentage [11,13]. Golzarand et al. [14] reported that vitamin D supplementation had no effect on body fat percentage. In contrast, Salepour et al. [15] showed that increasing vitamin D levels by supplementation led to body fat mass reduction. Therefore, whether vitamin D supplementation has an effect on body fat percentage especially in elite athletes remains unclear.

This prospective study was aimed to evaluate the effect of vitamin D supplementation on serum vitamin D level in elite male collegiate athletes and to analyze its effect on body fat percentage. The secondary aim of this study was to analyze the association between the change ratio of body fat percentage and change value of serum vitamin D level. We hypothesized that vitamin D supplementation would increase serum vitamin D level and would be related to lower body fat percentage. We also hypothesized that a negative correlation was observed between the change ratio of body fat percentage and change value of serum vitamin D level.

## Methods

### Participant selection

This study was approved by the Institutional Review Board and Ethics Committee of our institution before the study. A prospective analysis was performed.

The inclusion criteria for the participants were as follows: they should be collegiate athletes who are members of the national-champion-level teams in our institution and should be of the male sex. The exclusion criteria were as follows: (1) use of any medications or supplementation and (2) inability to participate in all examinations.

Participation was voluntary, and the participants were provided a thorough explanation of the objectives, methods, and ethical considerations of this study. Written informed consent was obtained from all participants included in the study.

### First examination

In March 2020, all athletes BMI, body fat percentage, and bone mass were measured using MC-980A-N plus (Tanita Corporation, Tokyo, Japan), a body composition analyzer.

Blood samples were collected to check the serum calcium, phosphorus, and 25(OH)D levels on the same day as the body composition monitoring. Serum 25(OH)D levels were analyzed using electrochemiluminescence immunoassay kits by SRL, Inc. (Tokyo, Japan). Serum calcium levels were measured through an enzymatic method using phospholipase D. Serum inorganic phosphate levels were measured through an enzymatic method using purine nucleoside phosphorylase.

### Supplementation and second examination

When all athletes were informed of the first examination results, they were provided vitamin D3 supplement tablets in the form of cholecalciferol 25g/day and Vitamin D Super 1000IU Nature Made by Otsuka Pharmaceutical Co. (Tokyo, Japan) to be taken for 3months. The use of the supplement was dependent on athletes preference without any force. Initially, the second examination similar to the first examination was scheduled 3months after the first examination. Unexpectedly, the coronavirus disease 2019 (COVID-19) outbreak occurred, and their club activities were banned for 2months from 1month after the first examination.

The second examination was conducted 1week after the ban on club activities was lifted. Body composition monitoring and blood test were performed similar to those in the first examination. Simultaneously, it was confirmed whether the supplement was used by each athlete.

The participants were divided into two groups according to vitamin D supplementation: the non-supplement group and the supplement group.

### Statistical analysis

All statistical analyses were performed with EZR (Saitama Medical Center, Jichi Medical University, Saitama, Japan), a graphical user interface for R (The R Foundation for Statistical Computing, Vienna, Austria) [16]. More precisely, it is a modified version of R Commander designed to add statistical functions frequently used in biostatistics. Students t*-*test and Fishers exact test were used to compare the data of the two groups. Paired t-test was used to compare the data of the first and second examinations in each group. Pearsons product moment correlation statistic was used to assess the correlation between the change ratio of body fat percentage (difference in the body fat percentage between the first and second examination) and the change in serum vitamin D levels (difference in serum vitamin D levels between the first and second examination). *P*-values<0.05 were considered statistically significant.

## Results

**Fig. 1:**
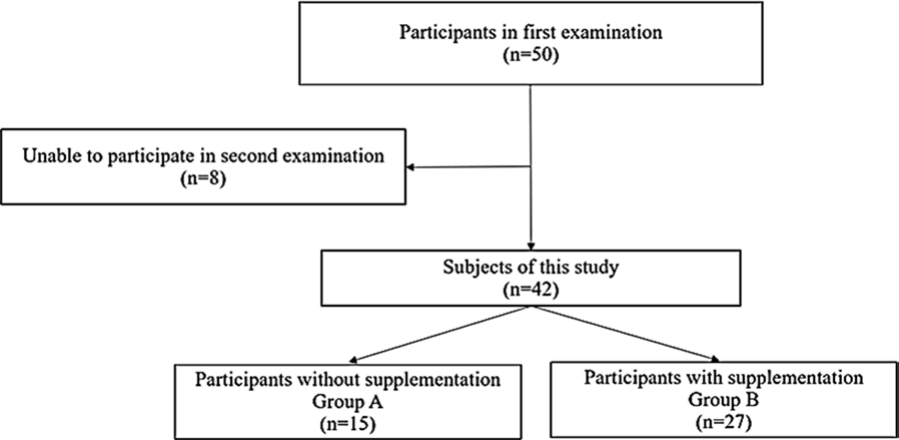
Flowchart of participant selection

Fifty elite male collegiate athletes participated in the first examination (Fig.1). Eight athletes were excluded because they could not participate in the second examination. Thus, 42 participants, including 24 hockey players and 18 fencing players, were included in the analysis: 15 participants in the non-supplement group and 27 participants in the supplement group.

In terms of baseline data of the first examination, there was a significant difference in the mean body fat percentage between the groups (Table 1; 9.0%1.9% and 12.1%3.8%, respectively; *P*=0.03). Moreover, the non-supplement group had a significantly higher serum 25(OH)D level than the supplement group (22.75.7 and 18.54.9ng/mL, respectively, *P*=0.02). There were no significant differences in participants age, height, BMI, bone mass, lean body mass, serum calcium level, and serum phosphorus level between the two groups.

**Table 1:** Baseline characteristics of the participants

	All athletes (n=42)	Non-supplement group (n=15)	Supplement group (n=27)	*P* value
Age, y	201	201	201	0.3
Height, cm	1725	1716	1725	0.7
Hockey/Fencing	24/18	11/4	13/14	0.2
*Body composition*				
BMI, kg/m^2^	21.42.6	20.41.1	22.03.0	0.06
Body fat percentage, %	11.03.5	9.01.9	12.13.8	**0.005**
Bone mass, kg	2.90.3	2.80.2	3.00.3	0.1
Lean mass, kg	56.15.0	54.53.7	57.15.4	0.1
*Laboratory results*				
Serum 25(OH) D, ng/mL	20.05.5	22.75.7	18.54.9	**0.02**
Serum Calcium, mg/dL	9.80.3	9.80.4	9.80.3	0.6
Serum Phosphorus, mg/dL	3.50.5	3.50.3	3.50.5	1.0

The values are given as meanstandard deviation

Bold indicates statistically significant *P* values (*P* < 0.05)

*BMI* body mass index

Significant differences were observed between the pre- and post-supplement BMI, body fat percentage, and serum 25(OH)D level in all athletes and the non-supplement group (21.42.6 and 22.02.7, respectively, *P*=0.04; 11.0%3.5% and 11.8%3.5%, respectively, *P*=0.04; 20.05.5 and 25.35.6ng/mL, respectively, *P*<0.001) (20.41.1 and 21.11.3, respectively; *P*<0.001, 9.0%1.9% and 10.9%2.8%, respectively; *P*=0.02, 22.75.7 and 24.45.5ng/mL, respectively, *P*=0.005) (Table 2). Moreover, significant differences were observed between the pre- and post-supplement serum 25(OH)D level in the supplement group (18.54.9 and 25.85.6, respectively; *P*<0.001). However, no significant differences were found between pre- and post-supplement BMI and body fat percentage.

**Table 2:** Pre and post BMI, body fat, and serum vitamin D level in each group

	Pre	Post	*P* value
*All athletes*			
BMI, kg/m^2^	21.42.6	22.02.7	**0.04**
Body fat percentage, %	11.03.5	11.83.5	**0.04**
Serum 25(OH) D, ng/mL	20.05.5	25.35.6	**>0.001**
*Non-supplement group*			
BMI, kg/m^2^	20.41.1	21.11.3	**>0.001**
Body fat percentage, %	9.01.9	10.92.8	**0.02**
Serum 25(OH) D, ng/mL	22.75.7	24.45.5	**0.005**
*Supplement group*			
BMI, kg/m^2^	22.03.0	22.53.1	0.2
Body fat percentage, %	12.13.8	12.33.8	0.9
Serum 25(OH) D, ng/mL	18.54.9	25.85.6	**>0.001**

The values are given as meanstandard deviation

Bold indicates statistically significant *P* values (*P* < 0.05)

*BMI* body mass index

In terms of follow-up data of the second examination, there were no significant differences in participants BMI, body fat percentage, bone mass, and lean body mass between the two groups (Table 3). There were also no significant differences in participants serum 25(OH)D level, calcium level, and phosphorus level between the two groups.

**Table 3:** Comparison between the groups after intervention

	All athletes (n=42)	Non-supplement group (n=15)	Supplement group (n=27)	*P* value
*Body composition*				
Post BMI, kg/m^2^	22.02.7	21.11.3	22.53.1	0.1
Change value of BMI, kg/m^2^	0.61.9	0.70.7	0.62.3	0.8
Post body fat percentage, %	11.83.5	10.92.8	12.33.8	0.2
Change ratio of body fat percentage, %	0.82.4	1.92.6	0.22.0	**0.02**
post Bone mass, kg	2.90.2	2.90.2	3.00.3	0.3
Change value of bone mass, kg	0.00.1	0.00.1	0.00.1	0.1
Post lean mass, kg	56.4.4.7	55.2.3.6	57.15.2	0.2
Change value of lean mass, kg	0.3.1.7	0.7.1.6	0.01.7	0.2
*Laboratory results*				
Post serum 25(OH) D, ng/mL	25.35.6	24.45.5	25.85.6	0.4
Change value of serum 25(OH) D, ng/mL	5.35.1	1.71.9	7.25.2	**>0.001**
Post serum Calcium, mg/dL	9.60.4	9.50.3	9.70.4	0.2
Change value of serum Calcium, mg/dL	0.10.5	0.30.6	0.10.4	0.2
Post serum Phosphorus, mg/dL	3.30.4	3.40.4	3.30.4	0.3
Change value of serum Phosphorus, mg/dL	0.20.6	0.10.5	0.20.6	0.4

The values are given as meanstandard deviation

Bold indicates statistically significant *P* values (*P* < 0.05)

*BMI* body mass index

In terms of change value from the first examination to the second examination, there were significant differences in body fat percentage between the groups (1.9%2.6% and 0.2%2.0%, respectively, *P*=0.02). Moreover, change value of serum 25(OH)D level in the non-supplement group was significantly lower than that in the supplement group (1.71.9 and 7.25.2ng/mL, respectively, *P*<0.001).

**Fig. 2:**
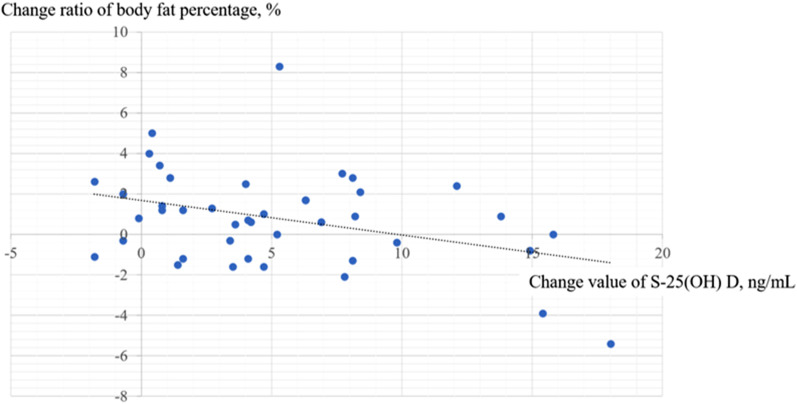
Correlation between the change ratio in body fat percentage and value in serum 25(OH)D level

A significant negative correlation was observed between the change ratio of body fat percentage and change value of serum 25(OH)D level (Fig.2, *r*=0.37, *P*=0.02).

## Discussion

This study might show the effectiveness of 25g/day vitamin D supplementation, which significantly increase the serum 25(OH)D level. Change ratio of body fat percentage in athletes without vitamin D supplementation was significantly higher than those in athletes with vitamin D supplementation under the unexpected situation of ban in club activities due to the COVID-19 outbreak. Moreover, a significant negative correlation was observed between the change ratio of body fat percentage and change value of serum 25(OH)D level in entire athletes in the same period.

Vitamin D has a significant impact on bone health, immune function, and physical performance. When there is deficiency, athletes may be at an increased risk of stress fractures, respiratory infections, and muscle injuries [17]. Female Navy recruits receiving vitamin D supplementation had a 20% lower incidence of stress fractures than recruits receiving placebo [2]. Therefore, vitamin D levels are greatly important for athletes, and vitamin D supplementation seems to improve vitamin-D-deficiency-related disorders in athletes.

Jung et al. [18] reported that 4weeks of 125g/day vitamin D supplementation increased the mean serum 25(OH)D level from 10.9ng/mL to 38.4ng/mL.Similarly, this study showed that vitamin D supplementation significantly increased the mean serum 25(OH)D level. However, the amount of daily vitamin D supplementation was 25g/day; therefore, the serum 25(OH)D level increase was milder than those in the previous study. Due to this mild increase, no sudden changes in blood calcium or phosphorus levels were observed.

Golzarand et al. [14] reported that vitamin D supplementation had no effect on body fat percentage. Conversely, Salepour et al. [15] reported thatincreasing 25(OH)D levels by vitamin D3 supplementation led to body fat mass reduction. In contrast, Renzo et al. [19] reported that the perception of weight gain was observed in 48.6% of the population during behavioral restriction due to the COVID-19 outbreak. During this study, a state of emergency was declared due to the COVID-19 outbreak, and sports activity was stopped, and athletes without supplementation gained body fat percentage of 1.9%. Conversely, athletes with supplementation had almost unchanged conditions and maintained their body composition, and as a result, there was a significant difference in the change value between the two groups.

Arunabh et al. [20] reported that the percentage of body fat is inversely related to the serum 25(OH)D level. Worstman et al. [21] explained that vitamin D insufficiency associated with obesity is due to decreased bioavailability of vitamin D from cutaneous and dietary sources due to its deposition in body fat compartments. However, it was reported that increasing vitamin D levels by supplementation led to body fat mass reduction in a randomized controlled trial [15]. Similarly, this study showed that vitamin D supplementation might be effective in maintaining body fat. Moreover, the percentage of body fat is inversely related to the serum 25(OH)D level in all the athletes included in the present study. Increased serum 25(OH)D level might suppress the increase in body fat percentage.

This study had several serious limitations. First, participants were not randomly assigned to the two groups. Therefore, selection bias might have influenced the outcomes. This may have also resulted in the differences in baseline body fat percentage and serum 25(OH)D levels between the two groups of participants. We believe this might be because of the lower serum 25(OH)D levels obtained on the first examination. More athletes might have taken the vitamin D supplement after they were informed of the first examination results. Moreover, body fat percentage is reported to be inversely related to serum 25(OH)D level [20]. These differences could have affected the change in the body fat percentage and the change in serum 25(OH)D level between the groups. Another possibility was that people who decided to take the supplement were in general healthier, since taking the supplement was voluntary, and could have had healthier lifestyles. Second, seasonal changes were not investigated. Maruyama-Nagao et al. [22] reported that athletes serum 25(OH)D levels were lower in March and higher in June. This might have also caused the increased serum 25(OH)D levels, even in the non-supplement group. Third, confirmation of the supplementation depended on each athletes self-report. This might have introduced errors and precise adherence was not determined. Fourth, the athletes food intake data were not analyzed. Food intake has an impact on the parameters measured and would have thus strongly affected the results of this study. Fifth, all the participants of the study were male. Body fat percentage is known to vary between female and male athletes. As this study design was not a randomized control study, only male athletes were included to avoid a big bias of sex difference. However, this would affect the generalizability of the results. Lastly, the sample size was relatively small.

## Conclusions

This study showed that 25g/day vitamin D supplementation significantly increased the serum 25(OH)D level in elite male collegiate athletes. Change ratio of body fat percentage in athletes without vitamin D supplementation were significantly higher than those in athletes with vitamin D supplementation under the unexpected situation of ban in club activities due to the COVID-19 outbreak. The percentage of body fat is inversely related to the serum 25(OH)D level. Vitamin D supplementation might have preferable effect on maintaining athletes body composition under circumstances where sports activity has decreased.

## Data Availability

The datasets used and/or analyzed during the current study are available from the corresponding author on reasonable request.

## Abbreviations

BMI: Body mass index
COVID-19: Coronavirus disease 2019
